# End-to-End Trained Energy-Based Model for Image Recovery

**DOI:** 10.3390/jimaging12090430

**Published:** 2026-09-10

**Authors:** Jyothi Rikhab Chand, Mathews Jacob

**Affiliations:** Department of Electrical and Computer Engineering, University of Virginia, Charlottesville, VA 22904, USA; mjacob@virginia.edu

**Keywords:** energy model, point estimate, memory efficient, MMSE estimate

## Abstract

This paper presents a novel end-to-end (E2E) empirical Bayes framework for learning the posterior distribution in linear inverse problems. The proposed framework models the log-posterior as the sum of a log-likelihood derived from the known forward model and a neural network-parameterized log-prior learned from training data. The approach enables both (a) computation of a point estimate i.e., stationary point of the learned log-posterior using a majorization minimization algorithm and (b) posterior sampling for computing the minimum mean square error (MMSE) estimate. Provided that the required surrogate function conditions are satisfied, the point estimation algorithm converges to a stationary point of the learned negative log-posterior without imposing contraction constraints. Unlike diffusion models that pre-learn the entire prior, learning the posterior directly leads to about 150× reduced training data requirements and 2× fewer parameters. Across three MRI acquisition settings, the framework improves point-estimation PSNR over PnP-ISTA by 3.07–4.37 dB while remaining competitive with existing E2E methods. Across two posterior sampling settings, it improves PSNR over DPS by 0.11–2.22 dB and over DAPS by 1.17–4.16 dB, while providing inference speedups of 2× and 5×, respectively. Furthermore, by avoiding algorithm unrolling, the proposed framework also reduces memory requirements by approximately 4× and 16× relative to E2E-MoL and E2E-EBM methods, respectively.

## 1. Introduction

Computational algorithms that can recover images from sparse and noisy measurements have revolutionized several fields, such as magnetic resonance (MR) imaging. Traditional compressed sensing (CS) methods typically compute a point estimate by minimizing an objective comprising a data-fidelity term and a hand-crafted image prior as the regularizer [1,2,3]. While effective, these priors often fail to capture complex image statistics, limiting performance at high accelerations.

Deep learning has greatly improved reconstruction. Plug-and-Play (PnP) methods replace CS proximal maps with convolutional neural network (CNN)-based denoisers [4,5,6] but require contraction constraints for convergence [7], which can reduce quality. Dropping these constraints help at low accelerations but risk instability at higher ones [8]. Energy-based PnP approaches address this by learning explicit energy functions, ensuring convergence without such constraints [9,10,11]. The energy model is learned using denoising score matching [9,10,11], maximum likelihood training [12,13,14,15], or distillation techniques [16]. Diffusion models learn an expressive time-dependent image prior to generate data consistent samples [17,18,19,20,21,22]. However, modeling the full image prior commonly requires large training datasets and time-conditioned networks with many parameters. Inference further requires long sampling trajectories, and obtaining the minimum mean square error (MMSE) estimate requires averaging multiple data consistent samples. Moreover, because these methods learn the score rather than an explicit scalar posterior energy, obtaining an optimization-based point estimate corresponding to a stationary point of the posterior energy is not straightforward.

Unlike the PnP approaches considered in the above paragraph, end-to-end (E2E) methods [23,24,25,26,27,28,29] often unroll the iterative recovery algorithm and optimize the CNN denoiser parameters so that the reconstructed image matches the reference image. Because E2E methods optimize the CNN parameters for a specific forward model, they often offer improved performance over PnP methods with smaller models and significantly less training data [26,30]. A challenge with current E2E models is the increased memory demand, resulting from algorithm unrolling. While methods such as deep equilibrium (DEQ) formulation [8,31,32] and checkpointing [33] strategies have been introduced, the former requires a contraction constraint and the latter trades memory efficiency with computational complexity. Meanwhile, energy-based DEQ methods, which do not require contraction constraints [28], are an attractive option in this setting. We note that these E2E approaches are typically designed to produce a single deterministic reconstruction and do not explicitly learn a posterior distribution from which samples can be generated.

Despite these advances, the methods considered above do not simultaneously provide acquisition-specific posterior learning, memory-efficient E2E training without algorithm unrolling, optimization-based point estimation, and posterior sampling. To address this gap, we propose Deep End-to-End Posterior ENergy (DEEPEN), a framework that directly learns an acquisition-specific negative log-posterior from the training data. We represent the log-posterior as the sum of a data-dependent log-likelihood term and the log-prior, which is represented using an energy model. The proposed approach is grounded in the empirical Bayes philosophy [34]. Unlike standard Bayes, that assumes the prior to be fixed a priori, we estimate the prior from training data by maximizing the data likelihood. Because the model parameters are optimized with respect to a task-level objective (log-likelihood) that depends on the measurements, we call DEEPEN an E2E approach to differentiate it from PnP methods that pre-train the energy function with a denoising task [12,13,14,15,35]. Once learned, the model parameters are fixed and used for all test instances, unlike hierarchical Bayes [36]. Our training strategy simplifies as the minimization of the posterior energy of the *true* reference samples, while maximizing the energy of the *fake* samples, which are generated using the Langevin sampling algorithm that utilizes the gradient of the posterior. This E2E training strategy is memory-efficient because it does not require unrolling as compared to most E2E methods. The proposed approach differs from [37], where their focus is to learn a regularizer that discriminates ground-truth images from the pseudo-inverse reconstruction. The learned function does not define a probabilistic model and is used solely as a regularization term within a variational reconstruction framework. In contrast, DEEPEN learns a probabilistic energy model which enables both point estimation and posterior samples from the modeled posterior distribution. Table 1 summarizes these distinctions.

The following are the main benefits of E2E-DEEPEN over PnP methods including diffusion models:

1. Effective with limited training data: Diffusion models require large datasets to model the prior distribution. In contrast, DEEPEN learns the posterior, conditioned on the forward operator using empirical Bayes. In this setting, less training data are sufficient to achieve comparable performance. For example, our model trained on 450 slices achieves performance comparable to that of a diffusion model trained on the full fastMRI dataset [38], making the proposed approach well-suited for data-scarce applications.
2. Efficient point estimation algorithm with stationary point guarantees: Clinical workflows often require a single image to be reconstructed rapidly. Our point estimation algorithm is ideally suited for this task; the computationally efficient point estimate offers performance comparable to the MMSE estimates given by the diffusion models, which require the generation of multiple posterior samples. In addition, the point estimation algorithm is guaranteed to converge to a stationary point of a cost function that approximates the learned log-posterior without contraction constraints on CNNs [9,10,11]. These guarantees are particularly valuable in medical imaging settings, where stability is critical (e.g., failures of ISTA when CNN constraints are relaxed). Combined with E2E training, this leads to improved performance and better generalization to unseen acquisitions compared to other E2E EBMs [28].

A preliminary conference version of this approach was previously published with limited results [39].

## 2. End-to-End Learned Energy Model

While the proposed method can be applied to general linear inverse problems, this paper focuses specifically on the reconstruction of MR images from undersampled measurements. Let $b\in C^{n}$ denote the noisy undersampled measurements from which we wish to recover the unknown image $x\in C^{m}$. The image and the measurements are related through a known linear forward operator $A\in C^{n\times m}$:

(1) b=Ax+n

where $n\in N(0,\eta^{2}I)$ is the additive complex white Gaussian noise. The recovery of the image from the measurements is often formulated as a maximum-a-posteriori estimation problem, where the solution is a maximum of the log-posterior:

(2) logp(x|b)=logp(b|x)+logp(x)

Here, p(x) is the prior distribution, and the log-likelihood term logp(b|x) is specified by:

(3) $$\log p\left(b|x\right)=-\frac{{∥Ax-b∥}^{2}}{2\eta^{2}}+P$$

where *P* is the normalization constant. The log-posterior can also be used to derive samples from the posterior distribution, which can inform about uncertainties in the estimation and offer likely solutions.

### 2.1. Deep End-to-End Posterior ENergy (DEEPEN)

We formulate the parametric model for the log-posterior distribution as:

(4) $$\log p_{\theta }\left(x|b\right)=\log p\left(b|x\right)+\log p_{\theta }\left(x\right),$$

where logp(b|x) is defined in (3) and $p_{\theta }\left(x\right)$ is an energy-based model (EBM) [35]:

(5) $$p_{\theta }\left(x\right)=\frac{1}{Z_{\theta }}\exp\left(-E_{\theta }\left(x\right)\right),$$

where $E_{\theta }\left(x\right):C^{m}\to R^{+}$ is modeled by a neural network and $Z_{\theta }$ is the normalization constant. Combining (3) and (5), we obtain the negative log-posterior $L_{\theta }\left(x\right)=-\log p_{\theta }\left(x|b\right)$ as:

(6) $$\begin{array}{ccc} L_{\theta }\left(x\right) & = & \underset{C_{\theta }\left(x;A,b\right)}{\underset{︸}{\frac{1}{2}{∥Ax-b∥}^{2}+E_{\theta }\left(x\right)}}+\log{\tilde{Z}}_{\theta }\left(b\right), \end{array}$$

where

(7) $${\tilde{Z}}_{\theta }\left(b\right)=\int e^{-C_{\theta }\left(x;A,b\right)}dx$$

is a normalization constant. For simplicity, we absorb the parameter $\eta^{2}$ into the definition of energy.

The common approach in EBMs is to pre-learn $p_{\theta }$ from fully sampled images, independent of the forward operator A [11,35,40]. Once the learning is complete, image recovery involves sampling from (4) using the learned $p_{\theta }$ or maximizing (4) as in [11]. We note that learning in such PnP methods is agnostic to the forward model A. However, methods based on E2E training (for example, [8,25,26,32]) often offer better performance than PnP methods. These E2E methods use pairs of measurements and images (x,b) to minimize the error between the reference image and the recovered image i.e., they try to learn the MMSE estimate. Motivated by the improved performance of E2E methods, we propose to train the EBM in an E2E fashion for a specific forward operator A in the next section.

### 2.2. Maximum Likelihood Training of DEEPEN

We determine the optimal weights of the parametric log-posterior by minimizing the negative log-likelihood of the training data set for a specific A operator:

(8) $$\begin{array}{ccc} \theta^{*} & = & \arg\min_{\theta }\;E_{x∼p(x)}E_{b∼p(b|x)}\left[-\log p_{\theta }\left(x|b\right)\right]\\ & = & \arg\min_{\theta }\;\underset{𝒥(\theta )}{\underset{︸}{E_{x,b∼p(x,b)}[-\log p_{\theta }\left(x|b\right)}}] \end{array}$$

Evaluating the gradient of the cost function in (8), we obtain:

(9) $$\begin{array}{c} \nabla_{\theta }𝒥\left(\theta \right)=E_{x,b∼p(x,b)}\left[\nabla_{\theta }C_{\theta }\left(x;A,b\right)+\nabla_{\theta }\log{\tilde{Z}}_{\theta }\left(b\right)\right]\\ \;\;\;\;\;\;\;\;\;\;\;\;\;=E_{x,b∼p(x,b)}\left[\nabla_{\theta }C_{\theta }\left(x;A,b\right)\right]+E_{b∼p(b)}\left[\nabla_{\theta }\log{\tilde{Z}}_{\theta }\left(b\right)\right]\\ \;\;\;\;\;\;\;\;\;\;\;\;\;=E_{b∼p(b)}E_{x∼p(x|b)}\left[\nabla_{\theta }C_{\theta }\left(x;A,b\right)\right]-E_{b∼p(b)}E_{x∼p_{\theta }\left(x|b\right)}\left[\nabla_{\theta }C_{\theta }\left(x;A,b\right)\right]. \end{array}$$

In the last step, chain rule is used to simplify $\nabla_{\theta }\log{\tilde{Z}}_{\theta }\left(b\right)$ for a fixed b using (7) as in [35] (for the sake of completeness we show the proof in Appendix A.1). Here, $x∼p_{\theta }\left(x|b\right)$ are termed *fake samples*, drawn from the parametric posterior distribution $p_{\theta }\left(x|b\right)$. For simplicity, we denote the fake samples as $x^{-}∼p_{\theta }\left(x|b\right)$, the reference samples corresponding to the measurement b are termed as *true samples*, denoted by $x^{+}∼p\left(x|b\right)$. We note that the ML training of energy models is now standard and has been used to pre-train the prior in PnP models [12,13,14,15,35]. By contrast, our main focus is the E2E training of the posterior sampling approach.

Thus, the ML estimation of θ is equivalent to minimization of the loss:

(10) $$\begin{array}{c} \;{𝒥}^{\prime }\left(\theta \right)=E_{b}\left(E_{x∼p(x|b)}\left[C_{\theta }\left(x;A,b\right)\right]-E_{x∼p_{\theta }\left(x|b\right)}\left[C_{\theta }\left(x;A,b\right)\right]\right)\\ \;\;\;\;\;\;\;\;\;\;\;\;\approx \frac{1}{m}\sum_{k=1}^{m}\left(\frac{1}{r}\left(\sum_{i=1}^{r}C_{\theta }\left(x_{i}^{+};A,b_{k}\right)-\sum_{j=1}^{r}C_{\theta }\left(x_{j}^{-};A,b_{k}\right)\right)\right) \end{array}$$

**Figure 1:**
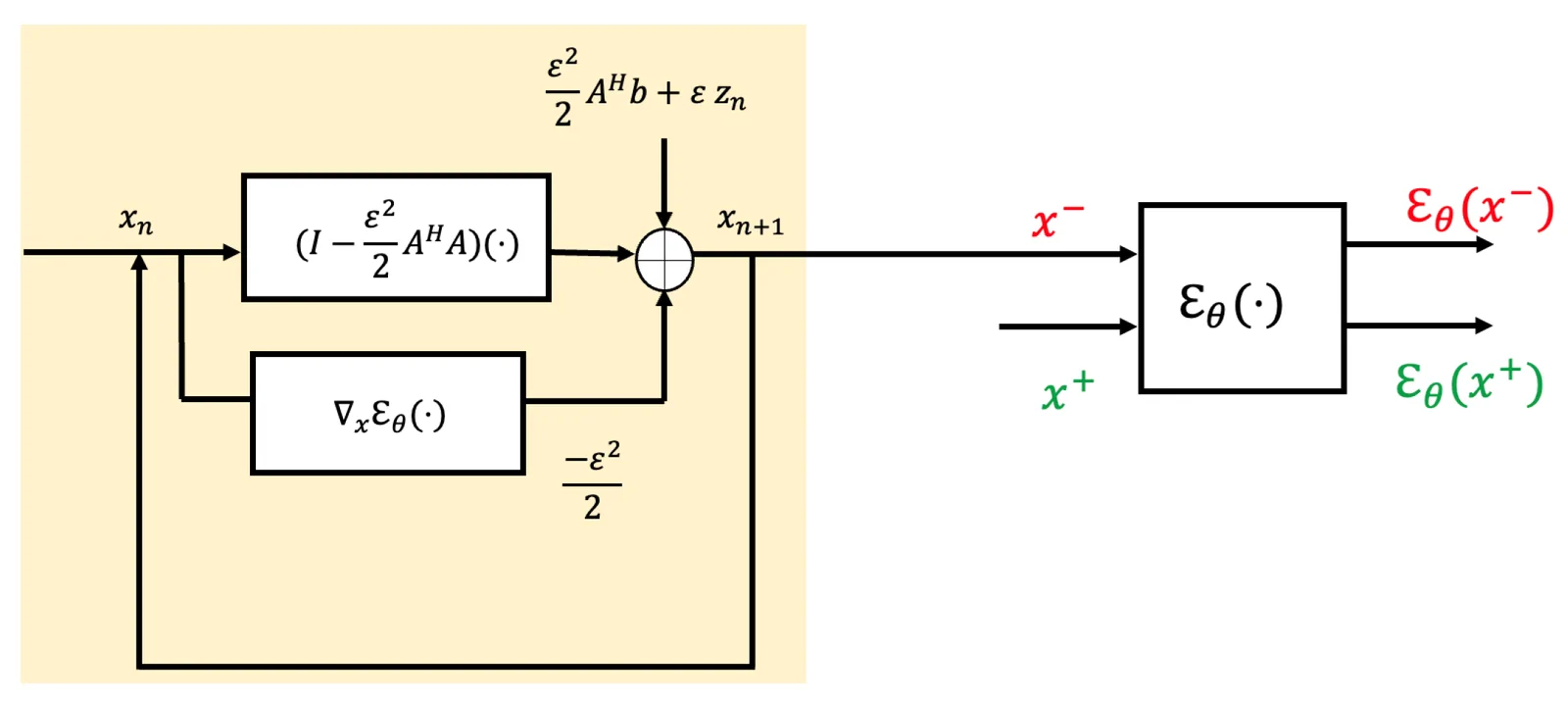
Demonstration of training procedure of DEEPEN. The training procedure determines the optimal weights of the energy $E_{\theta }\left(\cdot \right)$ by minimizing the energy difference between true and fake samples. The true samples $x^{+}$ are obtained from the training data, while the fake samples $x^{-}$ are generated using the Langevin sampling algorithm, highlighted by the yellow box. We note that the intermediate results are not stored to evaluate the loss’s gradient; therefore, a single physical layer is used for forward propagation. This keeps the training memory demand low.

Intuitively, the training strategy (10) aims to decrease the posterior energy of the true samples $C_{\theta }\left(x^{+};A,b\right)$ and increase the posterior energy of the fake samples $C_{\theta }\left(x^{-};A,b\right)$. Thus, this approach may be seen as an adversarial training scheme similar to [41], where $C_{\theta }$ serves as the classifier as shown in Figure 1. At the end of training, the energy of the fake samples are identical to that of the training samples; i.e, $C_{\theta }\left(x^{+};A,b\right)\approx C_{\theta }\left(x^{-};A,b\right)$. Unlike GAN models that use a separate generator to create the fake samples, the production of the fake samples in the proposed approach also relies on $C_{\theta },$ as described in the next subsection.

### 2.3. Generation of Samples from Posterior

We generate the fake samples $x^{-}∼p_{\theta }\left(x|b\right)$ using Markov Chain Monte Carlo (MCMC) methods. In particular, similar to [35], we use the Langevin MCMC algorithm which only requires the gradient of $\log p_{\theta }\left(x|b\right)$ w.r.t. x:

(11) $$\begin{array}{ccc} x_{n+1} & = & x_{n}-\frac{\epsilon^{2}}{2}\nabla_{x_{n}}C_{\theta }\left(x_{n};A,b\right)+\epsilon z_{n}\\ & = & x_{n}-\frac{\epsilon^{2}}{2}\left(A^{H}\left(Ax_{n}-b\right)+H_{\theta }\left(x_{n}\right)\right)+\epsilon z_{n}. \end{array}$$

Here, $H_{\theta }\left(x\right)\in \nabla_{x}E_{\theta }\left(x\right)$ is in the sub-differential of the energy. When $E_{\theta }$ is differentiable, $H_{\theta }\left(x\right)$ is the gradient. ϵ>0 is the step size, $z_{n}∼N\left(0,I\right)$, and $x_{0}$ is drawn randomly from a zero-mean Gaussian distribution. We note that $H_{\theta }\left(\cdot \right)$ does not depend on the normalization constant ${\tilde{Z}}_{\theta }$. The sampling algorithm in (11) is guaranteed to converge under regularity conditions [35]. Furthermore, since DEEPEN offers an expression for negative log-posterior, one may use faster sampling methods such as Metropolis-Hastings with larger step size. However, we do not consider it in this work.

### 2.4. Implementation of ML Loss (10)

We now consider the gradient of (10) w.r.t. θ for a fixed b:

(12) $$\begin{array}{c} \nabla_{\theta }{𝒥}^{\prime }\left(\theta \right)\approx \sum_{i=1}^{r}\nabla_{\theta }\;C_{\theta }\left(x_{i}^{+};A,b\right)-\sum_{j=1}^{r}{\nabla_{\theta }\;C}_{\theta }\left(x_{j}^{-};A,b\right)\\ \approx \sum_{i=1}^{r}\nabla_{\theta }\;E_{\theta }\left(x_{i}^{+}\right)-\sum_{j=1}^{r}{\nabla_{\theta }\;E}_{\theta }\left(\Omega \left(x_{j}^{-}\right)\right) \end{array}$$

where Ω is the stop gradient operator. Specifically, the gradients of $x_{j}^{-}$ in the second term with respect to θ are not considered in (12), which is consistent with the literature [35,41,42,43,44]. These ignored gradients are second-order corrections that add complexity without yielding empirical benefits (see Appendix A.2 for more details). In the second step, we used the fact that the posterior energy $C_{\theta }\left(\cdot \right)$ is separable in terms of the parameter-independent log-likelihood term and the parameter-dependent EBM. We note that the gradient in (12) implicitly depends on both A and b. In particular, the fake samples in (12) are generated using (11), which involves computing the gradient of $C_{\theta }\left(\cdot \right)$ w.r.t. x, that involves both A and b. Thus, the proposed method is trained in an E2E fashion.

For the point estimation experiments conducted in this work, DEEPEN was trained with 100 MCMC steps, initialized with Gaussian noise. Although energy models trained with similar short-run MCMC iterations have been reported to yield good results [12], a correction term [45] may be added to reduce bias. In addition, strict convergence of MCMC may not be needed for learning energy models, where the Langevin algorithm can be understood as learning a sampler instead [12]. Our experiments show that DEEPEN performs well, even with 100 steps. This may be due to the simpler structure of the posterior distribution (especially for low to moderate accelerations) when compared to the learning of the complex prior distribution considered in [45]. A similar improvement in convergence rate is also observed in [46], where the authors report good results (with as few as 10 MCMC steps) when they sample from a conditional distribution. The training procedure is summarized in Figure 1.

### 2.5. Point Estimation and Posterior Sampling for Image Recovery

Once training is complete, the images can be sampled from the posterior using Langevin sampling, specified by (11).

The proposed formulation also facilitates the computation of point estimate, where we minimize (6) with respect to x. In this work, we use the majorization minimization (MM) framework [47], which is guaranteed to converge monotonically [47,48] to a stationary point of (6). We note that any off-the-shelf optimization algorithm can be used to minimize (6). This is one of the key advantages of using EBMs, as they bridge the gap between compressed sensing and deep learning methods. The MM framework consists of two steps [47]. First, a surrogate function $g(x|x_{n})$ is constructed such that $L_{\theta }\left(x\right)\leq g\left(x|x_{n}\right)$. Next, the surrogate function is minimized to obtain the next iterate. In a similar fashion to [11], we used the following quadratic surrogate function to majorize $L_{\theta }\left(x\right)$ in (6):

(13) g(x|xn)=∥Ax−b∥22+Eθ(xn)+L2∥x−xn∥2+ReHθ(xn)H(x−xn)

where Re(·) considers the real part of the inner product, *L* is the Lipschitz constant of the trained score $H_{\theta }$ and can be approximately estimated using CLIP [49]. We note that we do not use any Lipschitz constraints on the architecture during training.

The above surrogate function has the following closed-form solution:

(14) $$x_{n+1}={\left(A^{H}A+LI\right)}^{-1}\left(A^{H}b+Lx_{n}-H_{\theta }\left(x_{n}\right)\right)$$

For a large-dimensional x, inverting the $\left(A^{H}A+LI\right)$ operator is computationally expensive. Therefore, we use the conjugate gradient algorithm to obtain the next iterate $x_{n+1}$. The following result [48] shows that the proposed algorithm converges monotonically to a stationary point of the cost function (6).

**Lemma** **1.** *Let $L_{\theta }:C^{m}\to R$ in (6) be bounded below, and consider the sequence $\left\{x_{n}\right\}$ generated by the MM update step (14). Suppose that, for every $x,y\in C^{m}$, the surrogate function satisfies*

(15) $$\begin{array}{cc} g(y|y) & =L_{\theta }\left(y\right),\\ g(x|y) & \geq L_{\theta }\left(x\right),\\ g_{x}^{\prime }\left(y|y;d\right) & =L_{\theta }^{\prime }\left(y;d\right),\;\forall \;d\in C^{m}. \end{array}$$

*and that g(x∣y) is jointly continuous in (x,y). Then every limit point $\bar{x}$ of $\left\{x_{n}\right\}$ is a stationary point of $L_{\theta }$ i.e., $L_{\theta }^{\prime }\left(\bar{x};d\right)\geq 0$ for every feasible direction d.*

**Proof.**  The result follows directly from Theorem 1 of [48].    □

**Figure 2:**
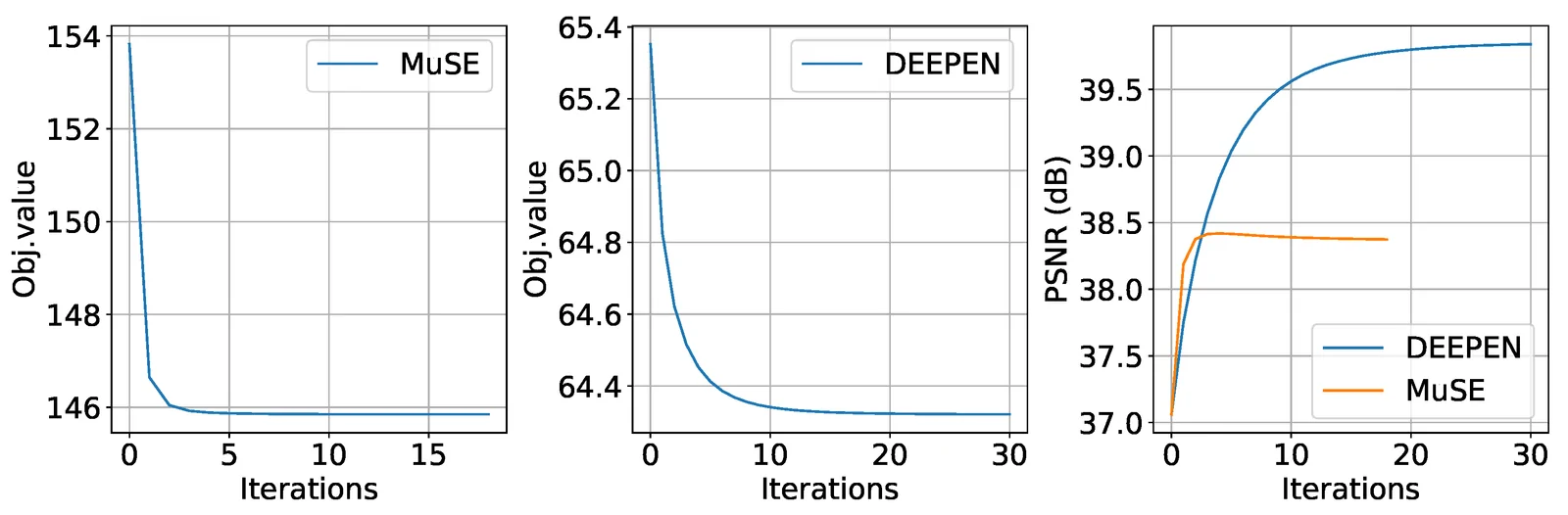
Convergence plot of energy models: MuSE and DEEPEN for four-fold 2D undersampling mask.

We note that the cost function $L_{\theta }\left(x\right)$ in (6) is a sum of negative log-likelihood and a learned energy function. We observe that the negative log-likelihood is lower bounded by zero. Furthermore, the CNN implementation of EBM can be lower bounded by using an appropriate output activation function (e.g., a quadratic activation in the output layer, which is considered in Section 4.1). Although an absolute-value output activation also ensures boundedness below, one or more of the surrogate conditions in (15) may be violated at its non-differentiable point, namely, when the input to the activation is zero. In practice, we observed a monotonic decrease in the cost function, as illustrated in Figure 2; however, this empirical monotonicity does not constitute a formal stationary point convergence guarantee for the absolute-value implementation.

## 3. Point Estimation Experiments & Results

We first compare DEEPEN with SOTA methods for point estimation. Training and inference is done on the A40 GPU (NVIDIA Corporation, Santa Clara, CA, USA).

### 3.1. Dataset

MR images were obtained from the publicly available 12-channel fastMRI brain dataset [38], consisting of complex images of size 320×320. The data set was divided into 45 training, 5 validation and 50 test subjects, each consisting of approximately 450, 50, and 500 images, respectively. We consider multichannel MR image reconstruction, where the forward operator is defined as A=SFC, where S is the sampling matrix, F is the Fourier matrix and C is the Coil Sensitivity Map (CSM) that is estimated using [50]. In this work, we consider Cartesian sampling pattern. We evaluated the models on T2-weighted images using 2D and 1D undersampling masks for different acceleration factors. We now describe the details of the proposed method as well as the SOTA methods. Note that the architecture of these methods takes a two-channel image (real and imaginary parts of the complex MR image) as input for energy or score calculation. Also, all the end-to-end methods considered in this work are trained for a specific acceleration and a sampling pattern.

### 3.2. DEEPEN & SOTA Methods for Point Estimation

We now describe the architectures of the methods used for point estimation.

#### 3.2.1. DEEPEN

We implement the energy model $E_{\theta }\left(x\right):C^{m}\to R^{+}$ as a CNN consisting of five 3×3 convolutional layers, followed by a linear layer. Each convolutional layer consists of 64 channels, and a Rectified Linear Unit (ReLU) was used between each layer, except the last linear layer. An absolute activation function was used in the linear layer to ensure that $E_{\theta }\left(\cdot \right)$ is lower bounded by a finite value. The score function $H_{\theta }\left(\cdot \right)$ was evaluated using the chain rule (PyTorch’s autograd library assumes that the derivative of ReLU at x=0 is zero, which corresponds to $H_{\theta }\left(x\right)\in \nabla_{x}E_{\theta }\left(x\right)$).

Similar to [51], we observe that the addition of Gaussian noise with zero-mean and standard deviation $2\epsilon^{2}$ to the training data stabilized the training. To improve training stability [51], we scale the posterior $p_{\theta }\left(x|b\right)$ by $\frac{\epsilon^{2}}{2}$, which gives rise to the equivalent Langevin MCMC update:

(16) $$\begin{array}{c} x_{n+1}=x_{n}-\left(A^{H}\left(Ax_{n}-b\right)+\nabla_{x_{n}}E_{\theta }\left(x_{n}\right)\right)+\epsilon z_{n} \end{array}$$

The MCMC Langevin algorithm was initialized with zero-mean Gaussian noise, step-size ϵ=0.001, and 100 MCMC sampling iterations were performed to generate the fake samples.

#### 3.2.2. PnP MuSE

We compare the proposed method with the MuSE energy model [11] that is trained in a PnP fashion. In particular, a single EBM is trained using multi-scale denoising score matching technique to predict Gaussian noise corresponding to a range of noise standard deviations. We used the following network architecture for MuSE:

(17) $$\begin{array}{c} E_{\theta }^{\mathrm{MuSE}}\left(x\right)=\frac{1}{2}{∥x-\psi_{\theta }\left(x\right)∥}^{2} \end{array}$$

where $\psi_{\theta }\left(x\right)$ is a five-layer convolutional network. Each layer was followed by ReLU activation, except in the final layer. The convolution layer consists of a 3×3 filter with 64 channels. MuSE was trained to predict Gaussian noise with standard deviations ranging from 0 to 0.1.

#### 3.2.3. PnP-ISTA

PnP-ISTA is motivated by the iterative soft thresholding approach [52] used in the CS algorithm. The proximal of the regularizer in the ISTA algorithm is replaced by a CNN denoiser, which is pre-trained as a Gaussian denoiser [53]. The optimization algorithm alternates between the following steps:

(18) $$\begin{array}{c} q_{t}=x_{t-1}-\alpha \frac{A^{H}\left(Ax_{t-1}-b\right)}{\eta^{2}} \end{array}$$

(19) $$\begin{array}{c} x_{t}=D_{\sigma }\left(q_{t}\right) \end{array}$$

where $D_{\sigma }\left(\cdot \right):C^{m}\to C^{m}$ is a CNN-based five-layer denoiser with each layer consisting of 64 channels with a 3×3 filter. Except for the final layer, after each layer the ReLU activation function was used. The denoiser was trained to remove Gaussian noise with standard deviation σ=0.01. To ensure convergence of the PnP-ISTA algorithm we imposed contraction constraints to the network (referred as PnP-ISTA-SN). This constraint was enforced using the spectral normalization technique. The performance of PnP-ISTA without the constraints is shown in the Supplementary Materials. As seen in [11] and in the Appendix A, PnP-ISTA (without contraction constraints) offers improved performance at lower accelerations but may translate to convergence issues and artifacts at higher acceleration rates.

#### 3.2.4. E2E MMSE Learning Using CNN Module

We also compare EBMs with an E2E approach MoL [8] that are trained to minimize MSE between recovered and reference images.

(20) $$\begin{array}{c} L_{\mathrm{MSE}}\left(\theta \right)=\frac{1}{2}\sum_{i=1}^{N_{\mathrm{samples}}}{∥x_{\theta }^{*}\left(i\right)-x_{\mathrm{ref}}\left(i\right)∥}^{2} \end{array}$$

Here, $N_{\mathrm{samples}}$ denotes the number of training samples, $x_{\mathrm{ref}}\left(i\right)$ is the reference image, and $x_{\theta }^{*}\left(i\right)$ is the solution to a regularized optimization problem that uses CNN as a regularizer [8]. The gradient of (20) is specified by

(21) $$\begin{array}{c} \nabla_{\theta }\;L_{\mathrm{MSE}}\left(\theta \right)=\sum_{i=1}^{N_{\mathrm{samples}}}\underset{l_{i}}{\underset{︸}{\left(x_{\theta }^{*}\left(i\right)-x_{\mathrm{ref}}\left(i\right)\right)}}\cdot \nabla_{\theta }x_{\theta }^{*} \end{array}$$

The DEQ approach [8,31,32] is used to derive the above gradient, which assumes the iterative algorithm to derive $x_{\theta }^{*}$ has converged to a fixed point. A monotone constraint is placed on the CNN to ensure convergence to the fixed point. We used a five-layer CNN to implement MoL and the monotone constraint was imposed using a log-barrier approach as described in [8].

#### 3.2.5. E2E Energy Models Using MSE Loss

The DEEPEN framework learns the posterior distribution using (8) in an E2E fashion, which can be used for posterior sampling or to derive point estimate. In contrast, ELDER [28] learns the regularizer parameters in an E2E fashion by minimizing the MSE loss (20), similar to the MoL approach described in the above section. We note that the ELDER algorithm that relies on DEQ strategy often converges rapidly. For simplicity, we consider the following iterative algorithm for K=30 iterations:

(22) $$\begin{array}{c} x_{k+1}=x_{k}-\alpha \left(A^{H}\left(Ax_{k}-b\right)+\nabla_{x_{k}}E_{\theta }\left(x_{k}\right)\right) \end{array}$$

to obtain the recovered image. Here, α is the step size and is a learnable parameter. We refer to our implementation as E2E-EBM (MSE).

Further implementation details and ablation studies are included in the Supplementary Materials.

### 3.3. Visualization of MuSE and DEEPEN Energies

In this section, we compare pre-trained MuSE with DEEPEN, whose energy model is trained in an E2E fashion. In particular, we evaluate the ability of the trained energy models to distinguish between clean image and noisy image: a well-behaved regularizer will assign lower energy to clean image and higher energy to noisy images. We evaluate the models for two different kinds of noise: (a) uncorrelated Gaussian noise z∼N(0,I) and (b) correlated structured noise, which approximates the difference between the original image and the least square solution (referred to as SENSE). To generate the structural perturbations, we compute the difference between the test image x and the SENSE solution $x_{0}={\left(A^{H}A+\tilde{\lambda }I\right)}^{-1}A^{H}b$. The motivation of choosing this particular structural perturbation is as follows: Point estimation based reconstruction methods are commonly initialized with SENSE, then the reconstruction algorithm must progressively reduce the structural error $s=(x_{0}-x)$. Accordingly the energy model must assign higher values to such perturbations to encourage the algorithm to suppress them.

**Figure 3:**
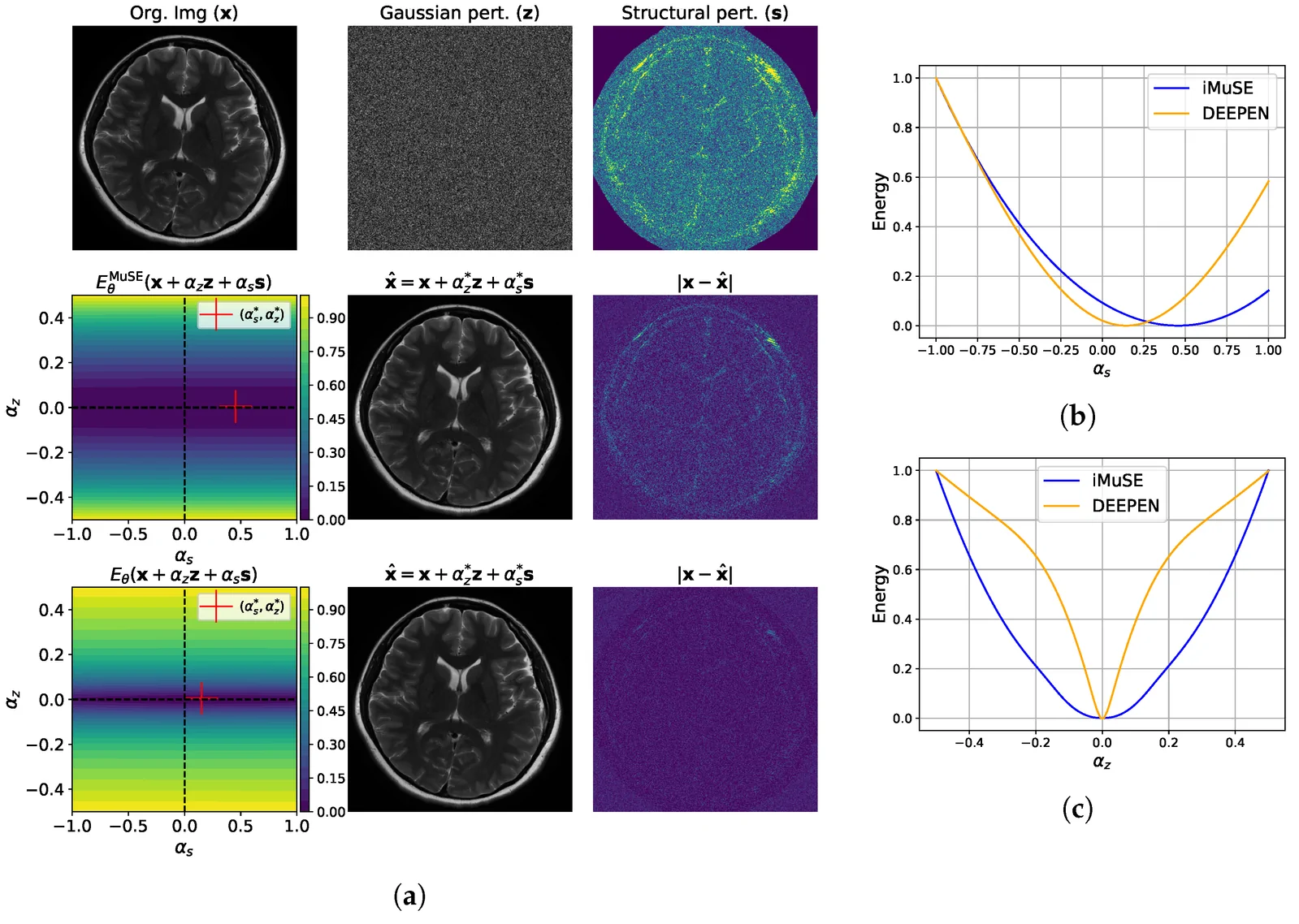
Comparison of pre-trained (MuSE) with E2E-trained (DEEPEN) energy models on six-fold acceleration. In (**a**), the top row shows the original image, Gaussian perturbation, and the structural perturbation specified by $x_{0}-x$, where $x_{0}$ denotes the SENSE solution; while the second and third row shows the 3D plot of MuSE and DEEPEN energy as a function of $\alpha_{z}$ and $\alpha_{s}$, their corresponding reconstructed and the error images, respectively. The images are reconstructed by taking the combination of the form $\hat{x}=x+\alpha_{s}^{*}s+\alpha_{z}^{*}z$, where $\left(\alpha_{s}^{*},\alpha_{z}^{*}\right)$ are the minimizer (indicated by cross mark in the contour plot) of the energy function. We note that the minimum of the DEEPEN energy is closer to $\alpha_{s}^{*}\approx 0;\alpha_{z}^{*}=0$, therefore the difference $|x-\hat{x}|$ is smaller than that of MuSE. In (**b**,**c**) we show the 2D plot of energy vs. Gaussian and structural perturbations, respectively. It can be observed that for the Gaussian perturbation, both energy models attain their minimum at $\alpha_{z}=0$; while for structural perturbations, DEEPEN’s minimum remains closer $\alpha_{s}\approx 0$, whereas MuSE reaches its minimum around $\alpha_{s}\approx 0.5$. This shows that the DEEPEN energy is effective in suppressing both correlated structural perturbations as well as Gaussian noise.

To assess this behavior, we feed images of the form $\tilde{x}=x+\alpha_{z}z+\alpha_{s}s$ to both energy models, where $\alpha_{s}$ and $\alpha_{z}$ vary from −1 to 1 and −0.5 to 0.5, respectively. An example of the test image along with Gaussian and structural perturbations is shown in the top rows of Figure 3a for six-fold acceleration. The second and third rows show the MuSE and DEEPEN energy contour plots as functions of $\alpha_{s}$ and $\alpha_{z}$, respectively. The red cross on the plot marks the minimizer $\left(\alpha_{s}^{*},\alpha_{z}^{*}\right)$ of the corresponding energy function. Ideally, a well-trained energy model should achieve its minimum at $\left(\alpha_{s}^{*},\alpha_{z}^{*}\right)=\left(0,0\right)$, reflecting the clean, unperturbed image. Additionally, Figure 3b,c show the 2D energy plots obtained by varying the Gaussian and structural perturbations independently.

We observe from Figure 3a that unlike MuSE, the energy model trained via DEEPEN has $\left(\alpha_{s}^{*},\alpha_{z}^{*}\right)$ closer to zero. This behavior is further reflected in the 2D energy plots in Figure 3b,c. For Gaussian perturbation (Figure 3b), both the energy models achieves minimum value at $\alpha_{z}^{*}=0$. However, when structural perturbations are added (Figure 3c), MuSE attains its minimum at $\alpha_{s}^{*}\approx 0.5$ while DEEPEN achieves its minimum value at $\alpha_{s}^{*}\approx 0$. We also show the image $\hat{x}$ evaluated at the minimizer $\left(\alpha_{s}^{*},\alpha_{z}^{*}\right)$, i.e., $\hat{x}=x+\alpha_{s}^{*}s+\alpha_{z}^{*}z$ and the corresponding error images in the second and third rows of Figure 3a. The error is computed by taking the absolute value of the difference between the test image x and $\hat{x}$. Note that since DEEPEN has $\left(\alpha_{s}^{*},\alpha_{z}^{*}\right)\approx \left(0,0\right)$, then the image $\hat{x}=x+\alpha_{s}^{*}s+\alpha_{z}^{*}z$ recovered by DEEPEN will be closer to the test image x. This is also reflected in the error maps, where MuSE has significant structural perturbations when compared to DEEPEN. This shows that DEEPEN is a better regularizer and, consequently, when employed in inverse problems, will be efficient in removing structural perturbations.

### 3.4. Reconstruction

In this section, we compare the reconstruction performance of DEEPEN with MuSE, E2E-EBM (MSE), PnP-ISTA-SN, and MoL. The energy-based models DEEPEN and MuSE were run until the cost function satisfied $|L_{\theta }\left(x_{n+1}\right)-L_{\theta }\left(x_{n}\right)\left|/\right|L_{\theta }\left(x_{n}\right)|\leq$$10^{-6}$ or until 500 iterations were reached. The hyperparameters for MuSE were the same as in [11]. We use the update equation in (22) as the inference algorithm for E2E-EBM (MSE) trained with MSE loss with K=30. PnP-ISTA-SN was run until $|x_{n+1}-x_{n}\left|/\right|x_{n}|\leq$$10^{-6}$ or until 500 iterations were reached.

All reconstruction algorithms were initialized with SENSE. Table 2 compares the reconstruction performance for three different settings: 4× and 6× acceleration with the 2D undersampling mask and 2× acceleration with 1D undersampling mask. The reconstructions are compared using two metrics: Peak Signal-to-Noise Ratio (PSNR) and Structural Similarity Index Measure (SSIM). Average PSNR and SSIM metrics are computed across slices.

**Figure 4:**
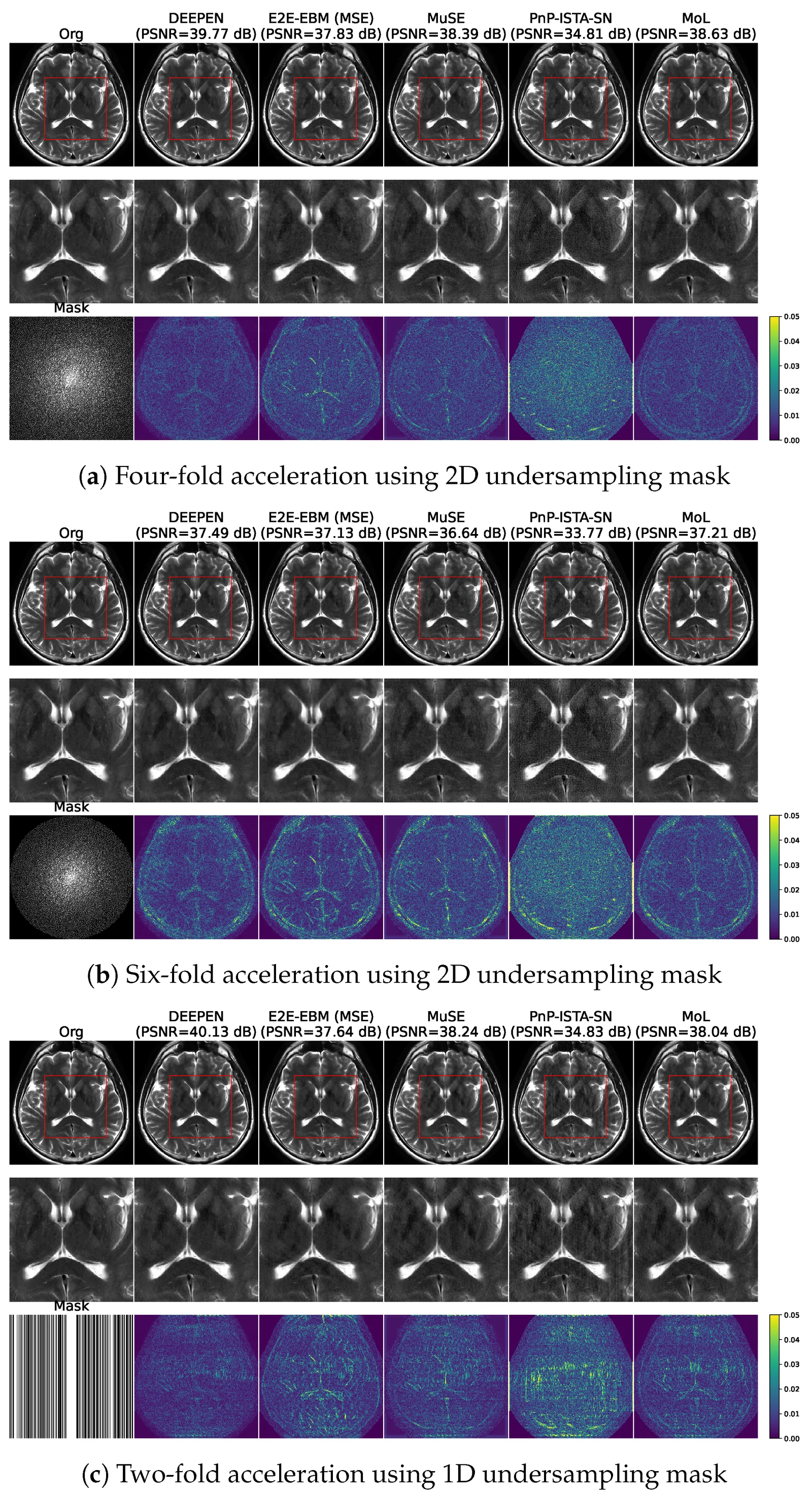
Comparison of DEEPEN, E2E-EBM (MSE), MuSE, PnP-ISTA, and MoL for three different acquisition settings on the fastMRI brain data set. The first, second, and the third row in each figure shows the reconstructed image, enlarged image, and the error image, respectively. The error image is scaled by a factor of 10 to highlight the differences.

We observe from Table 2 and Figure 4 that DEEPEN offers better results compared to MuSE at all acceleration settings. The improved performance of DEEPEN can be attributed to the E2E training strategy and its ability to suppress alias artifacts more effectively, as seen in Figure 3. We note that both energy models ensure convergence, without a contraction constraint on the score function, as discussed in Lemma 1. The convergence plot of DEEPEN and MuSE for the four-fold 2D undersampling mask shown in Figure 2 indicates that the cost functions monotonically decrease. PSNR plots show that while DEEPEN takes ≈20 iterations to converge compared to ≈ 6 iterations for MuSE, the E2E training strategy offers approximately a 1.2 dB improvement. The results also show that the DEEPEN approach also offers improved performance in comparison to MoL and E2E-EBM (MSE) in most cases. We note that the performance of DEEPEN is better than PnP-ISTA-SN across all the accelerations. We attribute this behavior to the contraction constraints required by the algorithm to guarantee convergence. In the absence of these constraints, the algorithm may diverge and produce artifacts in the reconstruction, as demonstrated in the Supplementary Materials.

### 3.5. Generalization Performance of E2E Energy Models

**Figure 5:**
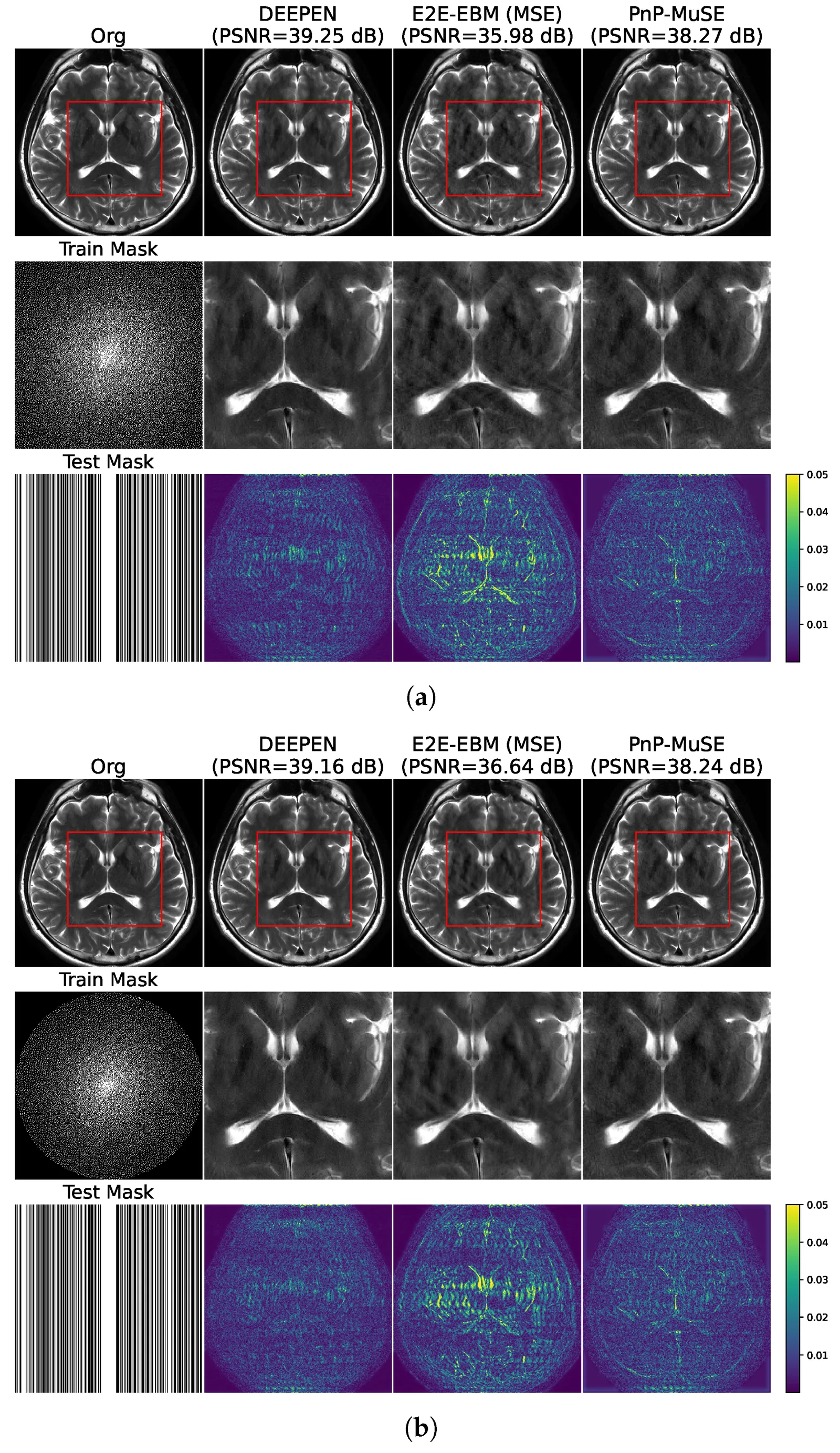
Generalization comparison of E2E-trained DEEPEN, E2E-EBM (MSE), and PnP-MuSE models for two different settings: (**a**) models are trained on 4-fold 2D undersampling mask and tested on a 2-fold 1D undersampling mask. When compared to Figure 4a, which demonstrates the reconstruction performance of models trained and tested on 4-fold 2D mask, the performance of DEEPEN and E2E-EBM (MSE) drops by 0.4 dB and 1.85 dB, respectively (**b**) models are trained on 6-fold 2D mask and tested on 2-fold 1D mask. When compared to Figure 4b, which demonstrates the performance of models trained and tested on 6-fold 2D mask, the performance of DEEPEN improved by 1.38 dB while the performance of E2E-EBM (MSE) dropped by 0.5 dB.

A challenge with unrolled E2E models that are trained for a specific forward model is their limitation in generalizing to other forward models (e.g., different acceleration rates). We now compare the generalization performance of DEEPEN and E2E-EBM (MSE), both of which are E2E energy models. The main difference is that DEEPEN learns the posterior using the maximum likelihood approach, while E2E-EBM (MSE) relies on MSE loss. We compare the generalization performance by considering a different acquisition scheme (sampling pattern) during the test setting from what was assumed during training. Table 3 compares the performance of DEEPEN and E2E-EBM (MSE) for different acquisitions. In the table, we report the average PSNR, where we highlight the performance of DEEPEN and E2E-EBM (MSE) in blue and black color, respectively. From the table, we observe that when there is a change in the acquisition setting, the performance of E2E-EBM (MSE) drops compared to DEEPEN in most cases. For example, when the energy models are trained on a 4-fold 2D undersampling mask and tested in the same setting, the performance of DEEPEN and E2E-EBM (MSE) is about 39.15 dB and 38.66 dB, respectively. However, when tested on a 2-fold 1D mask, the performance of E2E-EBM (MSE) drops by about 2.38 dB while the performance of DEEPEN drops only by 0.92 dB. Figure 5 illustrates the generalization performance of DEEPEN for two different mismatch scenarios. From Table 3, we also observe that DEEPEN trained in the 6× 2D undersampling mask performs marginally better when evaluated in the 4× 2D and 2× 1D sampling pattern. This improved performance may be due to the lower test acceleration. In the same table, we also report the performance of PnP-MuSE in red color. DEEPEN outperforms MuSE when the former is trained and tested in the same acquisition settings. However, when there is a mismatch, their performance is comparable in most settings.

We attribute the improved generalization performance of DEEPEN to the randomization of the paths in Langevin dynamics used to derive the fake samples. We note that Gaussian noise is added at every iteration of Langevin dynamics. The energy function is trained along different random paths that encourages the function to be well-defined around the solutions. In contrast, training with MSE loss learns a single path from the initialization and the reference image. We note that the iterations during inference depend on the energy function as well as the forward model; a change in the forward model can result in a different path to the solution, which may make E2E methods trained using MSE loss less robust to changes in acquisition settings.

### 3.6. Comparison of Training Memory for Different Algorithms

Current E2E methods often rely on unrolling, which translate to high memory demands (proportional to the number of iterations). By contrast, DEEPEN does not not unroll the Langevin iterations, and hence is significantly less memory demanding. Table 4 compares the training memory requirement of DEEPEN with other methods. Training memory requirements are computed for 50 slices and averaged over 10 epochs. For each epoch, the maximum memory usage was computed using the command torch.cuda.max_memory_allocated(). From the table it can be observed that the training memory requirement of DEEPEN per iteration (6-th column) is about four and thirteen times times lesser when compared to other E2E trained algorithms MoL and E2E-EBM, respectively.

## 4. Posterior Sampling Results

In this section, we compare DEEPEN with diffusion models in the context of posterior sampling. Training and inference is done on the A100 GPU (NVIDIA Corporation, Santa Clara, CA, USA).

### 4.1. DEEPEN and SOTA Architectures for Sampling

Here we compare the DEEPEN model with a pre-trained diffusion model in the context of posterior sampling [20]. We use a larger DEEPEN model compared to the ones used in the previous section to ensure fair comparisons.

#### 4.1.1. DEEPEN

We represent the energy $E_{\theta }\left(x\right)$ as:

(23) $$\begin{array}{c} E_{\theta }\left(x\right)=\frac{1}{2}{∥x-\phi_{\theta }\left(x\right)∥}^{2} \end{array}$$

where $\phi_{\theta }\left(\cdot \right)$ is a deeper DRUNet architecture that has four down and upscaling layers with 64, 128, 256, and 512 channels. Each downscaling and upscaling layer has four additional residual blocks. The total number of trainable parameters in DRUNet is about 32 million. Note that since scaling the Langevin algorithm (as in (16)) can affect the uncertainty estimate, we use the following unscaled annealed Langevin algorithm to train the parameters of the energy model with update steps:

(24) $$\begin{array}{c} x_{n+1}=x_{n}-\epsilon \left(A^{H}\left(Ax_{n}-b\right)+\nabla_{x_{n}}E_{\theta }\left(x_{n}\right)\right)+\sqrt{2\epsilon t}z_{n} \end{array}$$

where step-size ϵ=0.001, t>0 is the annealing temperature that starts with t=5 and decreases exponentially to t=0.002. At each epoch, 300 sampling iterations were performed and the algorithm was initialized with zero-mean Gaussian noise. We illustrate the evolution of fake samples during training in the Supplementary Materials.

#### 4.1.2. Diffusion Models

We used a pre-trained diffusion model, which uses a time-conditioned U-Net (We used the pretrained model from https://github.com/utcsilab/gsure-diffusion-mri, accessed on 27 March 2025) with approximately 65 million parameters. This network was trained using *EDM* hyperparameters, loss function, and noise schedule [19]. In particular, we used the following noise scheduler:

(25) $$\sigma_{i<N}={\left(\sigma_{max}^{1/\rho }+\frac{i}{N-1}\left(\sigma_{min}^{1/\rho }-\sigma_{max}^{1/\rho }\right)\right)}^{\rho },\;\sigma_{N}=0$$

where ρ=7, $\sigma_{max}=5$, $\sigma_{min}=0.002$. Diffusion posterior sampling (DPS) with zero-mean Gaussian initialization and 300 sampling steps were used to sample from the posterior [20]. We also compared DEEPEN with another diffusion-based algorithm: Decoupled Annealed Langevin Sampling (DAPS) [21]. To generate samples, DAPS with 100 Langevin steps, 5 ODE steps, and 200 annealing steps were used. We note that the DEEPEN model uses two times fewer parameters than the time-conditional diffusion model.

Most of the current E2E methods focus on learning the MMSE estimate [8,25,26,28]. While time-dependent diffusion models also provide the same, their long sampling chain makes their training in an E2E fashion difficult. We also note that it is challenging for diffusion models to realize a point estimate. In particular, diffusion models require the computation of the following complex integral to estimate the log-prior [18]:

(26) $$\log p_{\theta }\left(x\right)=\int_{0}^{T}\nabla .s_{\theta }\left(x\left(t\right),t\right)dt+\log p_{\pi }\left(x_{T}\right)$$

where $\log p_{\pi }\left(x_{T}\right)$ represents the final Gaussian noise distribution, $s_{\theta }\left(x\left(t\right),t\right)$ represents the time-conditional score model on scale *t*, and ∇ is the divergence operator. The DEEPEN framework offers the best of the above approaches, where the posterior distribution is learned in an E2E fashion and can be used to derive samples from the posterior, as well as offer point estimates.

### 4.2. Results

Table 5 compares the MMSE estimates obtained using DEEPEN and the diffusion model for two acquisition settings: 6× 2D and 4× 1D masks. The MMSE estimates are obtained by computing the mean of 30 generated samples. The average PSNR values are estimated across twenty subjects, and the table also reports the inference time (in seconds) required to generate a posterior sample. From the table it can be observed that for 6× 2D mask, DEEPEN achieves about 4.16 dB performance improvement when compared to DAPS and has similar reconstruction quality when compared to DPS. However, for the 4× 1D mask DEEPEN provides about 2.22 dB and 1.17 dB improvement when compared to DPS and DAPS, respectively. Notably, DEEPEN is approximately two and five times as fast as DPS and DAPS, respectively. This is because DEEPEN uses a smaller network when compared to the diffusion model. In particular, it uses two times fewer parameters. However due to the E2E training strategy, DEEPEN still achieves comparable performance.

**Figure 6:**
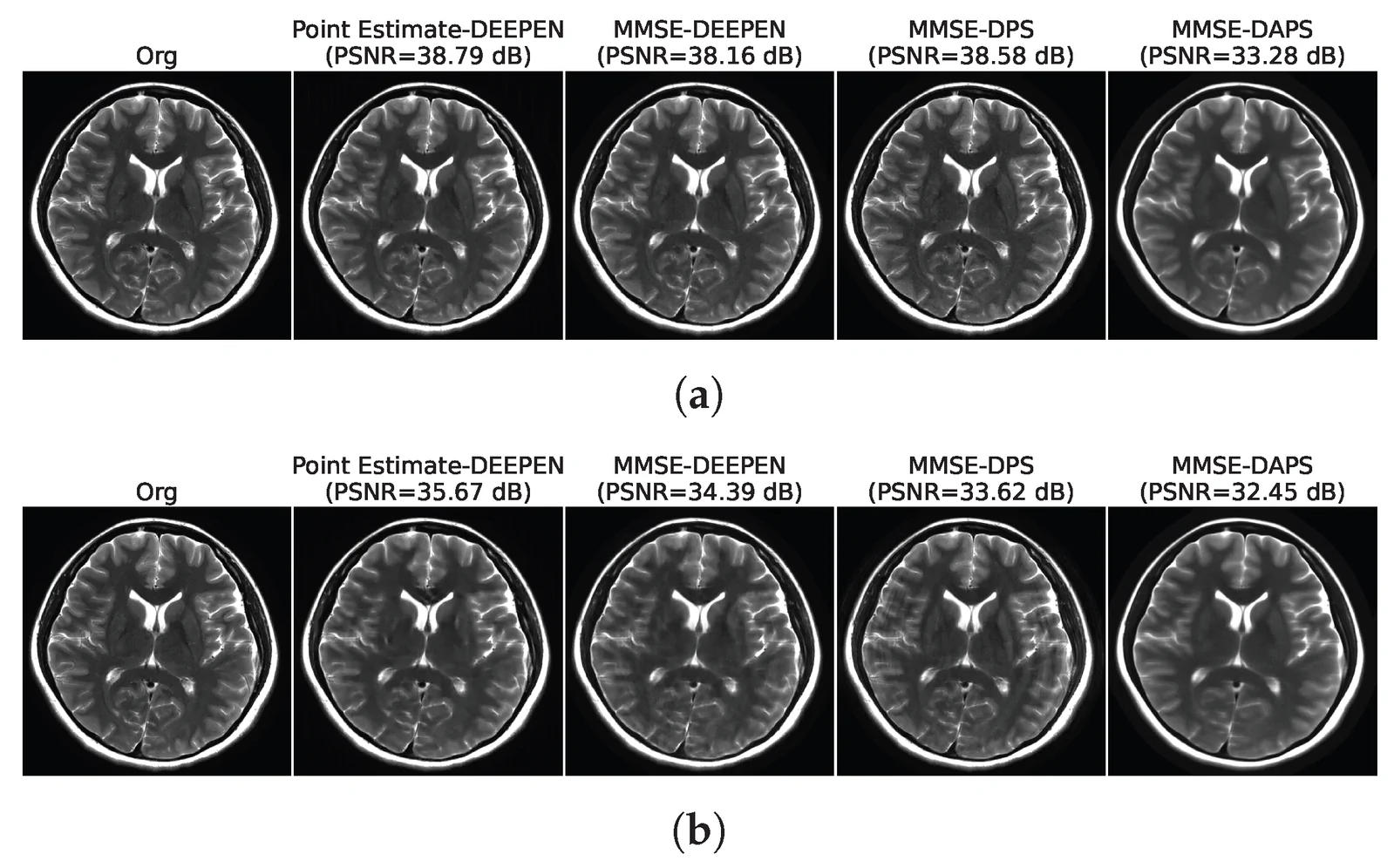
Comparison of DEEPEN (trained with 450 slices) with diffusion model (trained on entire fastMRI training data with multiple contrasts). (**a**) corresponds to 6× 2D undersampling mask and (**b**) shows the results for 4× 1D undersampling mask. Each row compares the MMSE provided by DEEPEN and diffusion based algorithms. In addition, we also show the point estimate given by DEEPEN. We observe that DEEPEN’s point estimate yields a higher PSNR compared to the MMSE estimate. This may be because the MMSE is approximated by averaging only 30 samples. We note that the DEEPEN network is smaller (has half the number of parameters of the diffusion model). Thanks to the smaller model, DEEPEN offers an approximate speedup of 2× and 5× when compared to DPS and DAPS algorithms, respectively.

Figure 6a,b compares the point and MMSE estimates provided by DEEPEN with the MMSE estimate of the diffusion models for 6× 2D and 4× 1D undersampling masks, respectively. In the case of diffusion models, we do not show the point estimate, since it is challenging to compute as discussed above. From Figure 6, we observe that in the 6× 2D setting, the MMSE and point estimates of DEEPEN and DPS are comparable. However, DEEPEN gives about 4.88 dB improvement in its MMSE recontruction when compared to DAPS. For a more challenging acquisition setting with a 4× 1D mask, DEEPEN gives about 0.77 dB and 1.94 dB improvement in its MMSE estimate compared to DPS and DAPS, while the point estimate is higher by 2.37 dB. We attribute the improved performance of DEEPEN to its E2E training strategy.

We note that one requires 300 iterations to generate a sample from the posterior distribution. To obtain a good approximation of MMSE, 30 such samples must be generated, resulting in 9000 iterations. However, obtaining a point estimate requires a maximum of 500 iterations (or even lesser). We acknowledge that there are faster diffusion-based posterior sampling algorithms [54], nonetheless to get MMSE estimate one still needs to generate several samples. Because the proposed point estimate is significantly more computationally efficient than the MMSE estimates offered by the diffusion models, the former may be preferable in many practical applications.

## 5. Discussion

DEEPEN is trained for a specific forward model A and is therefore not as generalizable as PnP and diffusion models. In contrast to diffusion models, which learn a general image prior, DEEPEN learns a task-specific posterior conditioned on the acquisition model. This specialization can improve parameter and data efficiency, but it also ties the model to a specific acquisition setting. Therefore, the comparison should be interpreted as a comparison between two different reconstruction paradigms rather than a controlled comparison that isolates the impact of architecture or model capacity alone. Generalization of DEEPEN can be improved by incorporating different masks during training. Furthermore, training involves generating fake samples via Langevin iterations which increases training run time. Faster sampling algorithms can be considered to reduce the training run time. Since DEEPEN learns the posterior distribution, it can also be used to obtain uncertainty maps. We consider analyzing DEEPEN’s uncertainty maps as part of future work.

## 6. Conclusions

We introduced DEEPEN, an E2E framework for learning posterior distributions in inverse imaging problems. By directly learning the posterior, DEEPEN enables both point estimation and posterior sampling within a unified framework. Unlike conventional unrolled E2E methods, DEEPEN does not unroll the Langevin iterations during training, resulting in lower memory requirements. Experimental results demonstrate that DEEPEN achieves competitive reconstruction quality while requiring fewer training samples than diffusion-based approaches and offering improved generalization to unseen acquisition settings compared to E2E energy models trained with an MSE objective. In addition, the proposed framework does not rely on contraction constraints to ensure convergence of the reconstruction algorithm. These results suggest that direct posterior learning is a promising and memory-efficient alternative for solving inverse imaging problems, particularly in data-limited settings.

## Figures and Tables

**Table 1:** Comparison of DEEPEN with other approaches. Parameter counts are reported in millions.

Algorithm	E2E	Forward Model Agnostic	Unrolled	Denoiser Constraint	Stationary Point Convergence	Point Estimate & Sampling	Parameter Count
DEEPEN	✓	×	×	×	✓	✓	6.70 (point estimation) 32.64 (posterior sampling)
MoL	✓	×	✓	✓	×	×	0.11
E2E-EBM (MSE)	✓	×	✓	×	×	×	6.70
PnP-ISTA-SN	×	✓	×	✓	×	×	0.15
MuSE	×	✓	×	×	✓	✓	0.15
Diffusion	×	✓	×	×	×	×	65.46

**Table 2:** Reconstruction performance of different models for three different acquisition settings.

Algorithm	2D 4× Mask		2D 6× Mask		1D 2× Mask	
	**Avg. PSNR**	**SSIM**	**Avg. PSNR**	**SSIM**	**Avg. PSNR**	**SSIM**
DEEPEN	39.15 ± 1.43	0.98 ± 0.007	37.18 ± 1.32	0.97 ± 0.009	38.91 ± 2.21	0.98 ± 0.01
MuSE	38.27 ± 1.30	0.97 ± 0.007	36.64 ± 1.20	0.96 ± 0.007	38.37 ± 1.88	0.97 ± 0.01
PnP-ISTA-SN	34.94 ± 1.34	0.94 ± 0.01	34.11 ± 1.25	0.93 ± 0.016	34.54 ± 1.61	0.95 ± 0.018
MoL	38.12 ± 1.27	0.97 ± 0.007	36.91 ± 1.19	0.97 ± 0.008	37.72 ± 1.67	0.97 ± 0.01
E2E-EBM (MSE)	38.66 ± 1.23	0.98 ± 0.005	37.39 ± 1.17	0.97 ± 0.006	38.02 ± 1.52	0.97 ± 0.008

**Table 3:** Generalization performance of E2E-trained energy model: DEEPEN (in blue), E2E-EBM (MSE), and PnP-MuSE (in red). The best performing model is boldfaced.

		Train Setting		
		**4× 2D Mask**	**6× 2D Mask**	**2× 1D Mask**
Test setting	4× 2D mask	**39.15 ± 1.43** 38.66 ± 1.23 38.27 ± 1.30	37.60 ± 1.53 **38.88 ± 1.24** 38.27 ± 1.30	**38.64 ± 1.55** 37.86 ± 1.20 38.27 ± 1.30
	6× 2D mask	**37.50 ± 1.29** 36.82 ± 1.18 36.64 ± 1.20	37.18 ± 1.32 **37.39 ± 1.17** 36.64 ± 1.20	**37.20 ± 1.37** 36.26 ± 1.14 36.64 ± 1.20
	2× 1D mask	38.23 ± 1.99 36.28 ± 1.43 **38.37 ± 1.88**	37.66 ± 1.98 36.54 ± 1.46 **38.37 ± 1.88**	**38.91 ± 2.21** 38.02 ± 1.52 38.37 ± 1.88

**Table 4:** Comparison of training memory requirements.

Algorithm	End-to-End	CNN Weight Constraints	No. of Iterations (MCMC/Unrolling) During Training	Memory Req. (MB)	Memory Req/Iteration (MB)
DEEPEN	Yes	No	100	5172.36	51.72
E2E-EBM	Yes	No	30	20,827.84	694.26
MoL	Yes	Yes	10	2273.4	227.34
MuSE	No	No	-	1550.94	-
PnP-ISTA	No	No	-	933.87	-

**Table 5:** Sampling performance comparison of DEEPEN and diffusion on two different accelerations.

Algorithm	Avg. PSNR ± Std		Inference Time (s)
	**4× 1D Mask**	**6× 2D Mask**	
DEEPEN	32.41 ± 1.88	37.07 ± 1.27	10.66
DPS	30.19 ± 2.19	36.96 ± 1.49	24.31
DAPS	31.24 ± 2.27	32.91 ± 1.41	56.15

## Data Availability

The original data presented in the study are openly available in https://fastmri.med.nyu.edu (accessed on 27 March 2025).

## Supplementary Materials

The following supporting information can be downloaded at: https://www.mdpi.com/article/10.3390/jimaging12090430/s1, Figure S1: (a) compares the PSNR distribution of DEEPEN with PnP-ISTA and PNP-ISTA-SN (b) illustrates examples where the PnP-ISTA algorithm diverges and produces bright artifacts in the reconstructions, which disappear only when additional constraints are imposed on the score network; Figure S2: Evolution of fake samples as a function of epochs for 4× 1D acceleration; Figure S3: Impact of (a) Langevin step-size ϵ and (b) Langevin iterations on the reconstruction performance of DEEPEN and energy-based model training independent of the forward model using the maximum likelihood (ML) loss (c) training data size.

## Appendix A

### Appendix A.1. Gradient of *log*${\tilde{Z}}_{\theta }$ w.r.t. θ

From (7) we know that:

(A1) $$\nabla_{\theta }\log{\tilde{Z}}_{\theta }=\nabla_{\theta }\log\int e^{-C_{\theta }\left(x;A,b\right)}dx$$

Applying chain rule we get:

(A2) $$\begin{array}{c} \nabla_{\theta }\log{\tilde{Z}}_{\theta }={\left(\int \exp^{-C_{\theta }\left(x;A,b\right)}dx\right)}^{-1}\nabla_{\theta }\left(\int \exp^{-C_{\theta }\left(x;A,b\right)}dx\right)\\ ={\left(\int \exp^{-C_{\theta }\left(x;A,b\right)}dx\right)}^{-1}\left(\int \nabla_{\theta }\exp^{-C_{\theta }\left(x;A,b\right)}dx\right) \end{array}$$

The above can be further simplified as:

(A3) $$\begin{array}{c} {\left(\int \exp^{-C_{\theta }\left(x;A,b\right)}dx\right)}^{-1}\left(\int \nabla_{\theta }\exp^{-C_{\theta }\left(x;A,b\right)}dx\right)\\ =-{\left(\int \exp^{-C_{\theta }\left(x;A,b\right)}dx\right)}^{-1}\\ \left(\int \exp^{-C_{\theta }\left(x;A,b\right)}\nabla_{\theta }C_{\theta }\left(x;A,b\right)dx\right)\\ =-\int {\left(\int \exp^{-C_{\theta }\left(x;A,b\right)}dx\right)}^{-1}\\ \left(\exp^{-C_{\theta }\left(x;A,b\right)}\nabla_{\theta }C_{\theta }\left(x;A,b\right)dx\right) \end{array}$$

Using (6) and (7) we get:

(A4) $$\begin{array}{c} =-\int {{\tilde{Z}}_{\theta }}^{-1}p_{\theta }\left(x|b\right){\tilde{Z}}_{\theta }\nabla_{\theta }C_{\theta }\left(x;A,b\right)dx\\ =-\int p_{\theta }\left(x|b\right)\nabla_{\theta }C_{\theta }\left(x;A,b\right)dx\\ =-E_{x∼p_{\theta }\left(x|b\right)}\nabla_{\theta }C_{\theta }\left(x;A,b\right) \end{array}$$

### Appendix A.2. Gradient of Fake Samples w.r.t. θ

Consider the posterior energy:

$$C_{\theta }\left(x;A,b\right)=\frac{1}{2}{∥Ax-b∥}^{2}+E_{\theta }\left(x\right),$$

which depends on the forward model A and the measurement b. The fake samples are generated using *N* steps of Langevin sampling algorithm:

(A5) $$x_{n+1}=x_{n}-\frac{\epsilon^{2}}{2}\left(\nabla_{x_{n}}{∥Ax_{n}-b∥}^{2}+\nabla_{x_{n}}E_{\theta }\left(x_{n}\right)\right)+\epsilon z_{n}$$

where ϵ is step-size and $z_{n}∼N\left(0,I\right)$. Note that the above update step depends on θ only through $\nabla_{x}E_{\theta }\left(x\right)$. The exact gradient of the training objective:

$$\nabla_{\theta }J^{\prime }\left(\theta \right)=E\left[\nabla_{\theta }C_{\theta }\left(x^{+};b\right)\right]-E\left[\nabla_{\theta }C_{\theta }\left(x_{N};b\right)\right]$$

which is expanded as:

(A6) $$\begin{array}{c} \nabla_{\theta }{𝒥}^{\prime }\left(\theta \right)=\underset{\mathrm{positive}\;\mathrm{phase}}{\underset{︸}{E[\nabla_{\theta }E_{\theta }\left(x^{+}\right)]}}-\underset{\mathrm{direct}\;\mathrm{negative}}{\underset{︸}{E[\nabla_{\theta }E_{\theta }\left(x_{N}\right)]}}-\\ \underset{\mathrm{path}\;\mathrm{term}}{\underset{︸}{E\left[{\left(\nabla_{x_{N}}C_{\theta }\left(x_{N};b\right)\right)}^{⊤}\frac{\partial x_{N}}{\partial \theta }\right]}}. \end{array}$$

We now consider the term $\frac{\partial x_{N}}{\partial \theta }$ in the path term. Each Langevin step depends on $\nabla_{x}E_{\theta }\left(x\right)$, so differentiating $x_{n+1}$ w.r.t. θ gives:

$$\frac{\partial x_{n+1}}{\partial \theta }=\frac{\partial x_{n}}{\partial \theta }-\frac{\epsilon^{2}}{2}\underset{=\nabla_{\theta x_{n}}^{2}E_{\theta }\left(x_{n}\right)}{\underset{︸}{\left(\frac{\partial }{\partial \theta },\nabla_{x_{n}},E_{\theta },\left(x_{n}\right)\right)}}+(\mathrm{chain}\;\mathrm{rule}\;\mathrm{terms}).$$

Thus, the path term involves $\nabla_{\theta x}^{2}E_{\theta }\left(x\right)$, the mixed second-order derivative of the energy function. Ignoring this term removes all second-order contributions, leaving only first-order derivatives $\nabla_{\theta }E_{\theta }\left(x\right)$ in the update. Stopping gradients through the Langevin algorithm during θ-updates is equivalent to assuming that negative samples approximate the posterior under the current θ, capturing first-order dependence on A and b. The ignored terms are second-order corrections that add complexity without significant empirical benefit.
