# Supplementary material for: End-to-End Trained Energy-Based Model for Image Recovery
Source: J Imaging. 2026 Sep 10;12(9):430. doi: 10.3390/jimaging12090430 (PMC13608472; doi:10.3390/jimaging12090430)
Supplement: Supplementary file 1 [file jimaging-12-00430-s001.zip › jimaging-4466292-supplementary.pdf]

Supplementary Materials

End-to-End Trained Energy-Based Model for Image Recovery

Jyothi Rikhab Chand * and Mathews Jacob

Department of Electrical and Computer Engineering, University of Virginia, Charlottesville, VA 22904, USA; mjacob@virginia.edu
* Correspondence: jyothi-rikhabchand@virginia.edu

S1. Performance comparison of PnP-ISTA (with and without constraints) with DEEPEN

Figure S1a compares the PSNR distribution of DEEPEN, PnP-ISTA, and PSP-ISTA-SN (where we enforce spectral normalization constraints on the score network) on the 6× 2D undersampling mask. From the figure, it can be observed that PnP-ISTA has several outliers when compared to DEEPEN. Nonetheless, the algorithm converges when constraints are added to the network but has poorer performance. In Figure S1b, we also illustrate some of the reconstructions where PnP-ISTA fails and has artifacts in its recovery when compared to DEEPEN and PnP-ISTA-SN.

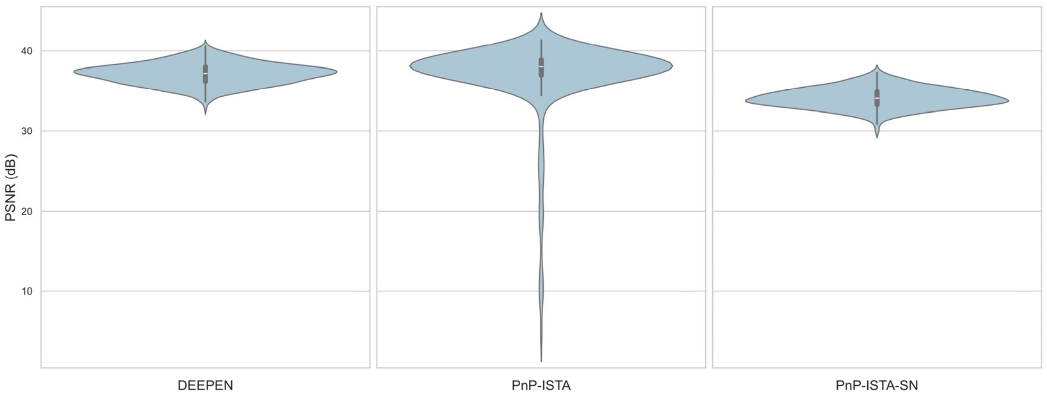

(a)

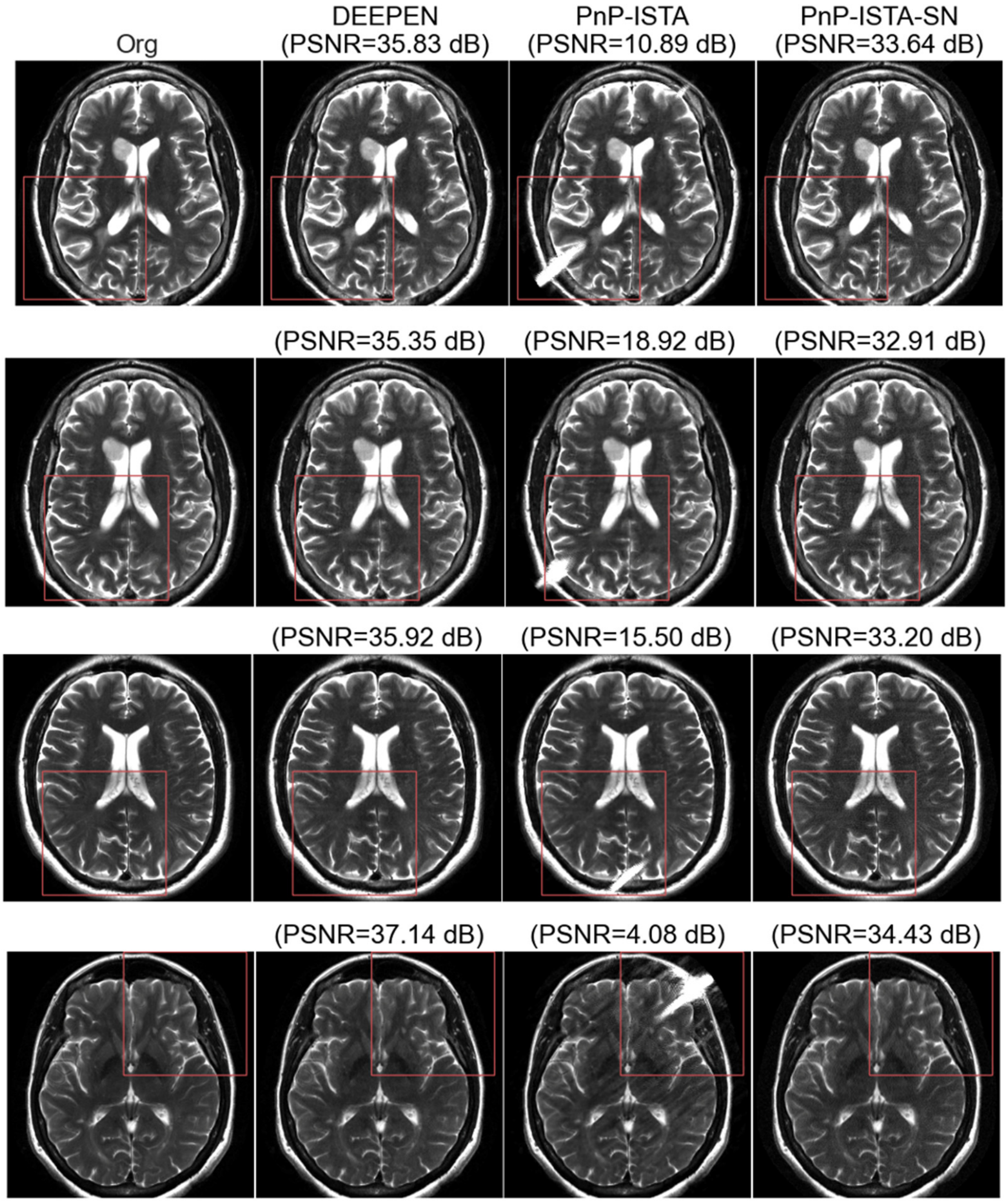

(b)

Figure S1. (**a**) compares the PSNR distribution of DEEPEN with PnP-ISTA and PNP-ISTA-SN (**b**) illustrates examples where the PnP-ISTA algorithm diverges and produces bright artifacts in the reconstructions, which disappear only when additional constraints are imposed on the score network.

S2. Evolution of fake samples during training

Figure S2 shows the evolution of fake samples as a function of epochs when DEEPEN is trained for the A operator corresponding to the 4× 1D sampling mask. As can be seen from the figure, the PSNR gradually improves over the epochs. In the figure, we overlay three fake samples that were generated corresponding to epochs 30, 60, and 150. The fake samples were generated by initializing the Langevin algorithm randomly from a zero-mean Gaussian distribution and running the algorithm for 300 iterations.

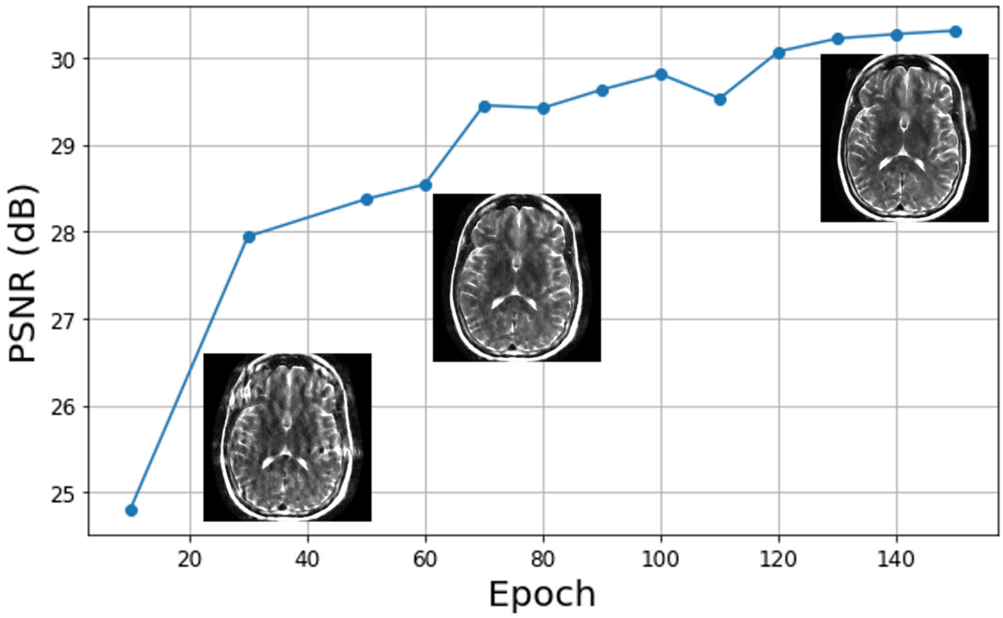

Figure S2. Evolution of fake samples as a function of epochs for 4× 1D acceleration.

S3. Ablation studies

Figure S3a shows the effect of training DEEPEN with different Langevin step sizes on reconstruction performance. Specifically, three DEEPEN models were trained with step sizes of 0.001, 0.01, and 0.1, with the number of Langevin iterations fixed at 100. All three models were trained for the acquisition setting corresponding to 2D mask and 4× acceleration. As shown in the figure, the recovery performance is comparable for step sizes 0.001 and 0.01; however, performance drops by approximately 6.51 dB at a step size of 0.1.

Next, four DEEPEN models were trained with Langevin iterations of 10, 30 , 50, and 100, with the step size fixed at 0.001. These models were trained for the 2D mask 6× acceleration setting. Figure S3b shows the effect of varying the number of Langevin iterations on reconstruction performance, which improves as the number of iterations increases. Additionally, four plug-and-play energy-based models (PnP-EBM) (corresponding to Langevin iterations of 10, 30, 50, and 100) were trained using the maximum likelihood loss, independent of the forward operator A. We observe a similar trend as with DEEPEN; but more importantly, the end-to-end (E2E) training strategy enabled DEEPEN to achieve better performance compared to PnP-EBM that is trained independently of the forward operator.

Figure S3c shows the impact of varying the number of training data on the MMSE reconstruction performance for 4× 1D acceleration. Three DEEPEN models were trained on 100, 300, and 450 slices with same architecture, Langevin iterations, and step size. From the figure it can be observed that when the model is trained with 100 and 300 training slices the performance only drops by 0.5 dB and 0.16 dB, respectively. This is a direct consequence of training DEEPEN in an end-to-end fashion, which learns the conditional posterior distribution, as opposed to diffusion models, which learn the unconditional prior distribution-thereby making them data hungry.

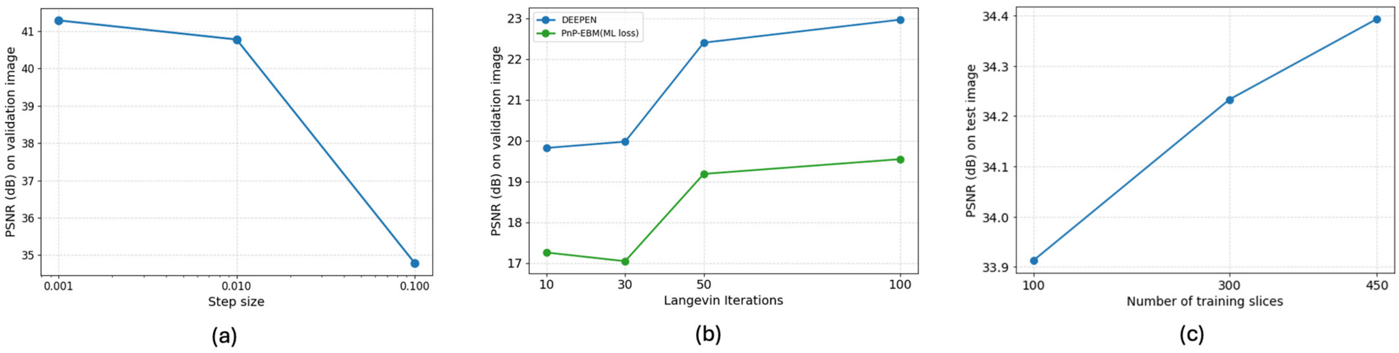

Figure S3. Impact of (**a**) Langevin step-size ϵ and (**b**) Langevin iterations on the reconstruction performance of DEEPEN and energy-based model training independent of the forward model using the maximum likelihood (ML) loss (**c**) training data size.

S4. Implementation details

Normalization involved scaling the k-space data such that the magnitude of the images were less than one. For DEEPEN, no data-fidelity weight was used. As stated below Eq. (7) in the main paper, the data-fidelity weight η^2 was absorbed into the definition of the learned energy $E_\theta(x)$. Consequently, DEEPEN's data-fidelity term contains only a fixed coefficient of 0.5. For the competing methods, the data-fidelity parameter were selected using the validation set. The 2D 4× and 6× masks were generated using nonuniform variable-density Poisson-disc sampling. The 2D 4× mask contained 25,460 acquired locations out of 102,400 Cartesian k-space locations, corresponding to a sampling density of 24.86% and an effective acceleration of 4.02×. The 2D 6× mask contained 17,037 acquired locations out of 102,400, corresponding to a sampling density of 16.64% and an effective acceleration of approximately 6.01×. The 1D 2× Cartesian mask contained 26 contiguous fully sampled central low-frequency lines. The remaining phase-encoding lines were selected randomly to obtain the specified acceleration. The generated masks were stored and used consistently for all comparison methods. The parameters of the energy model were optimized using the Adam optimizer with a batch size equal to four. In the case of MAP experiments with 2D undersampling masks, the learning rate was fixed as 1×10^{-5}. For the 1D mask 2× acceleration, the same learning rate with *ReduceLRonPlateau* scheduler was utilized. For the sampling experiments, irrespective of the undersampling mask,

learning rate of 1×10^{-7} with *ReduceLRonPlateau* scheduler was used to train the model. 100 and 200 epochs were used to train DEEPEN for MAP and sampling experiments, respectively. No global random seed was fixed during model training. We saved every 10th epoch model during training and selected the best model among them for inference using the validation set which produced the lowest reconstruction error.
